# Water use efficiency in a primary subtropical evergreen forest in Southwest China

**DOI:** 10.1038/srep43031

**Published:** 2017-02-20

**Authors:** Qing-Hai Song, Xue-Hai Fei, Yi-Ping Zhang, Li-Qing Sha, Yun-Tong Liu, Wen-Jun Zhou, Chuan-Sheng Wu, Zhi-Yun Lu, Kang Luo, Jin-Bo Gao, Yu-Hong Liu

**Affiliations:** 1 Key Laboratory of Tropical Forest Ecology, Xishuangbanna Tropical Botanical Garden, Chinese Academy of Sciences, Menglun 666303, China; 2 Global Change Ecology Group, Xishuangbanna Tropical Botanical Garden, Chinese Academy of Sciences, Menglun 666303, China; 3 University of Chinese Academy of Sciences, Beijing 100049, China; 4 Ailaoshan Station for Subtropical Forest Ecosystem Studies, Jingdong 676200, Yunnan, China

## Abstract

We calculated water use efficiency (WUE) using measures of gross primary production (GPP) and evapotranspiration (ET) from five years of continuous eddy covariance measurements (2009–2013) obtained over a primary subtropical evergreen broadleaved forest in southwestern China. Annual mean WUE exhibited a decreasing trend from 2009 to 2013, varying from ~2.28 to 2.68 g C kg H_2_O^−1^. The multiyear average WUE was 2.48 ± 0.17 (mean ± standard deviation) g C kg H_2_O^−1^. WUE increased greatly in the driest year (2009), due to a larger decline in ET than in GPP. At the diurnal scale, WUE in the wet season reached 5.1 g C kg H_2_O^−1^ in the early morning and 4.6 g C kg H_2_O^−1^ in the evening. WUE in the dry season reached 3.1 g C kg H_2_O^−1^ in the early morning and 2.7 g C kg H_2_O^−1^ in the evening. During the leaf emergence stage, the variation of WUE could be suitably explained by water-related variables (relative humidity (RH), soil water content at 100 cm (SWC_100)), solar radiation and the green index (Sgreen). These results revealed large variation in WUE at different time scales, highlighting the importance of individual site characteristics.

## Long-term water use efficiency (WUE)

Ecosystem WUE indicates the coupling of carbon and water vapor flux exchanged between the atmosphere and an ecosystem and it quantifies how much water an ecosystem uses relative to carbon gained^1^,^2^. The trade-off between the amount of carbon assimilated and the amount of water transpired has been examined widely in fields ranging from plant physiology to irrigation and afforestation science^3^,^4^,^5^. At the leaf level, stomata regulate the relation between carbon assimilation and water transpiration. At the ecosystem level, water availability is the primary limiting factor in carbon sequestration^6^,^7^. Given ongoing climate change, a deeper understanding of ecosystem WUE will improve the ability to predict carbon and water cycles^2^,^6^,^7^,^8^,^9^.

The temporal dynamics of WUE differ strongly depending on plant types and climate conditions^10^,^11^. Therefore, consistent and continuous observations for accurate evaluations of WUE can provide insight into how the ecosystems respond, or have responded, to climate fluctuations at different temporal scales, from hourly to multiannual^10^,^12^. However, most long-term characteristics of WUE in forest ecosystems are measured using the tree-ring carbon isotope method^8^,^13^. Tree-ring isotopes have the advantage of recording long-term changes, but may not be reliable in quantifying the responses of WUE to local climate change. Currently, there are limited studies on direct long-term WUE analysis^2^,^14^. For example, in one study the observed increase in forest WUE was most consistent with a strong CO_2_ fertilization effect^2^. In another study, no inter-annual trend was detected in WUE in a tropical rainforest, either annually or seasonally^14^. Nevertheless, the long-term patterns of WUE have not yet been well quantified in other forests^15^.

## Ailaoshan forest

The Ailaoshan Nature Reserve in Yunnan province, southwestern China hosts about 5000 ha of primary subtropical evergreen mountain cloud forest. This area is exposed to monsoon precipitation (P) regimes from the southwest and from the southeast. Due to the effect of the two monsoons, the climate in the study region is strongly seasonal with a wet season and a dry season^16^. A widespread and severe drought occurred in southwestern China in 2009 and 2010, providing a unique opportunity to directly evaluate how WUE changes with drought stress in the primary subtropical forest.

## Environmental and biological control on WUE

Previous studies in the nature reserve based on continuous, automated camera monitoring have shown clear seasonal patterns of canopy phenology. Automated image analysis (greenness index, Sgreen) has provided reliable information on developmental stages of the dominant tree species through all seasons. Variations in plant phenology affect the phase, timing, and magnitude of ecosystem carbon sequestration and hydrological processes^17^,^18^,^19^,^20^. Therefore, the impact of vegetation phenology modification (the timing of leaf emergence, developmental, and senescence stages) on WUE is likely to be critical.

## Drought impact on WUE

WUE has been recognized as an effective trait for assessing ecosystem response to climate change^21^. Drought affects the carbon balance by modifying both the rates of carbon uptake by photosynthesis (GPP) and release by ecosystem respiration, and the coupling between them^9^. Most studies of anomalous drought have shown that drought-induced tree mortality may severely weaken the carbon budget of forest ecosystems. However, another study showed a consistent increase in biomass in western African dry forests, indicating that specific community composition may play a key role in allowing the carbon stocks of these forests to be maintained during drought periods^22^. In terms of the global average, “warmer is less arid” from meteorological, hydrologic and agro-ecological perspectives, at least when that warming is induced by elevated CO_2_^23^. Whatever, the drought will simultaneously produce a deep decrease in evapotranspiration (ET), thereby leading to uncertain WUE.

## Specific objectives of this study

This study analyzed eddy-covariance (EC) measurements of carbon and water vapor exchange from 2009 to 2014. Specific objectives were to:

1. Characterize the instantaneous, seasonal and inter-annual variability of WUE in the subtropical evergreen forest.
2. Explore environmental and biological controls of WUE; and
3. Quantify the degree of impact of WUE by drought stress.

### Study site

**Figure 1:**
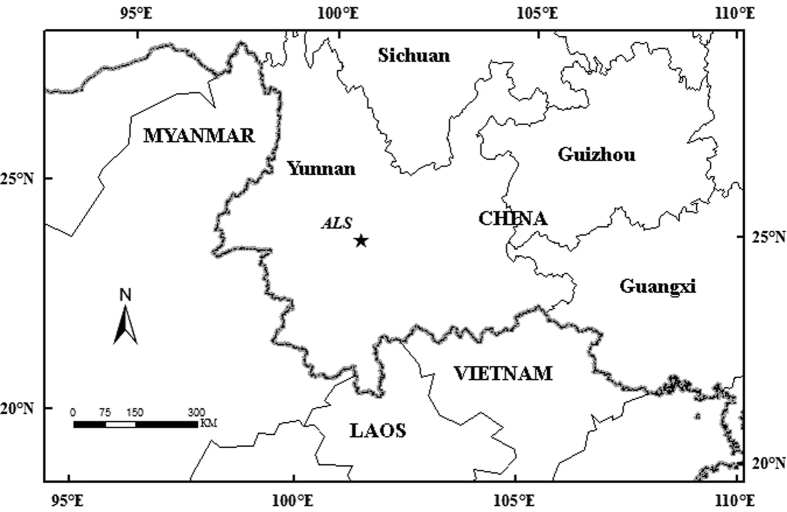
The location of the study site (ALS: Ailaoshan). The figure was created using Arcgis 8.2 software (ESRI Inc., Redlands, CA) (http://www.esri.com/software/arcgis/arcgis-for-desktop).

The study area was in the Xujiaba region of southwestern China in the Ailaoshan National Nature Reserve at 24°32′N, 101°01′E and 2476 m above mean sea level (Fig. 1). It lies within a protected section of a 5100 ha evergreen forest with a stand age of more than 300 years^24^. The forest is primarily dominated by *Lithocarpus hancei* (Benth.) *Rehder, Machilus bombycina, Castanopsis rufescens* (Hook.f.et Th.) *Huang* et Y.T. Chang, and *L (Lithocarpus). Xylocarpus*^25^. The mean canopy height was 20 m (Figure S1). The soils are loamy Alfisols, and the 3–7 cm organic carbon horizon had a pH of 4.5^26^,^27^.

## Results

### WUE and UWUE patterns at multiple scales

**Figure 2:**
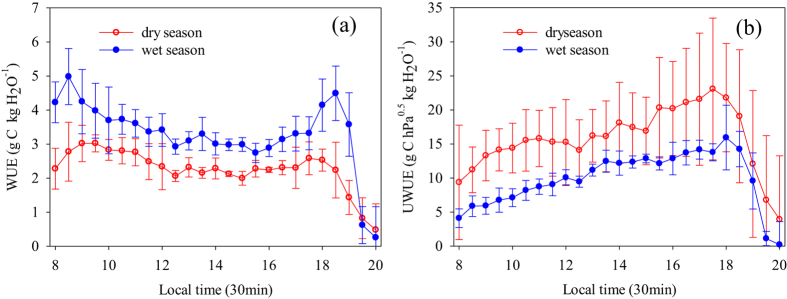
The diurnal dynamic of water use efficiency (WUE; **a**) and underlying water use efficiency (UWUE; **b**) with 30 min data in dry and wet season.

The diurnal, seasonal and inter-annual variations of WUE and UWUE were evaluated. Instantaneous WUE during the wet and dry season, which was estimated every half hour, showed a varied diurnal cycle trend, with a primary WUE maximum in the early morning and a secondary maximum in the evening (Fig. 2). WUE in the wet season reached 5.1 g C kg H_2_O^−1^ in the early morning and 4.6 g C kg H_2_O^−1^ in the evening. WUE in the dry season reached 3.1 g C kg H_2_O^−1^ in the early morning and 2.7 g C kg H_2_O^−1^ in the evening. WUE in the wet season was higher than that in the dry season in the daytime. In contrast, UWUE in the wet season was lower than that in the dry season. Unlike the diurnal dynamic of WUE, the UWUE reached a peak in the evening (between 17:00 and 18:00) and then decreased sharply.

**Figure 3:**
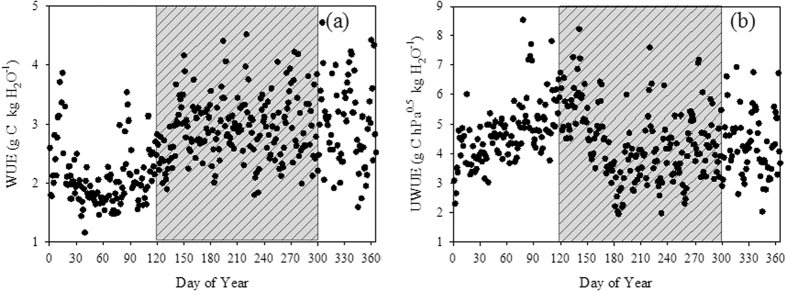
Annual variability of WUE (**a**) and UWUE (**b**) (grey bars indicate the wet season).

The annual cycles of WUE and UWUE are shown in Fig. 3. WUE exhibited considerable seasonal variation, with the highest values during the wet season and the lowest values during the later dry season (February and March) (Fig. 3a). In contrast, UWUE was highest in March (8.54 g C hPa^0.5^ kg H_2_O^−1^) and lowest in July (1.95 g C hPa^0.5^ kg H_2_O^−1^) (Fig. 3b).

**Figure 4:**
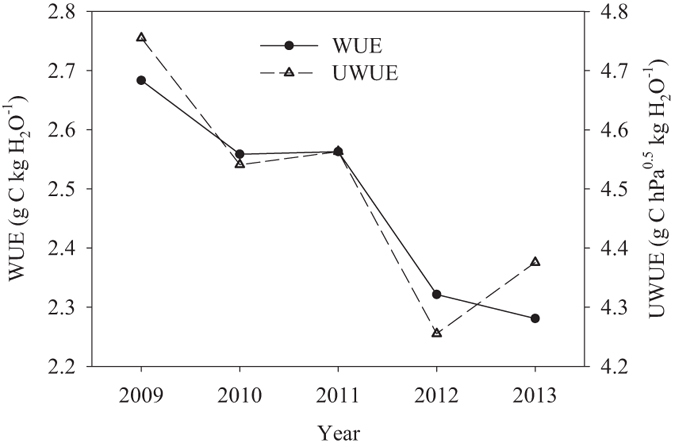
Inter-annual variation of water use efficiency (WUE) and underlying water use efficiency (UWUE).

Annual mean WUE exhibited a decreasing trend from 2009 to 2013, which varied from ~2.28 (2013) to 2.68 g C kg H_2_O^−1^ (2009) (Table 1 and Fig. 4). The multiyear average WUE was 2.48 ± 0.17 (mean ± standard deviation) g C kg H_2_O^−1^. WUE was greatly increased in 2009, the driest year. We further discuss the effect of drought stress on WUE in the following section. UWUE showed a similar trend with WUE from 2009 to 2012. However, UWUE in 2013 increased (Table 1 and Fig. 4).

At the annual scale, by examining the GPP and ET of each year, it can be seen that highest WUE in 2009 was caused by more deceased ET and lowest WUE in 2013 was caused by more deceased GPP (Table 1).

### Influence of environmental controls on WUE

To explore the drivers and limiting conditions of WUE under the varying climatic conditions in more depth, we statistically evaluated the correlations of WUE with its potential drivers.

**Figure 5:**
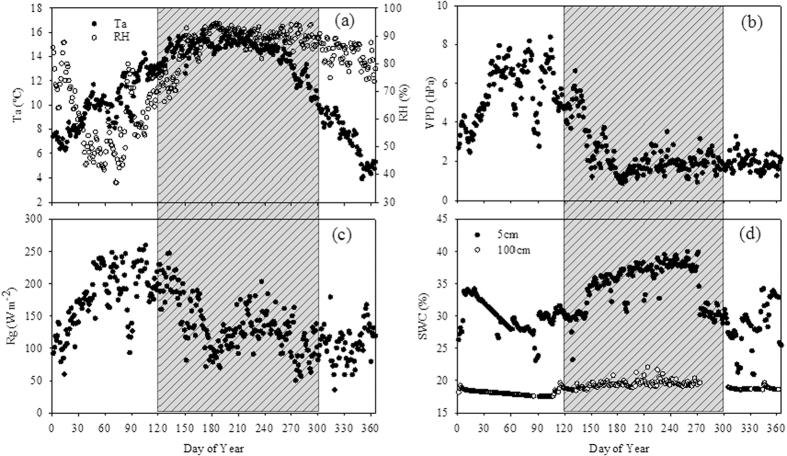
Annual cycles of (**a**) air temperature (Ta) and relative humidity (RH), (**b**) water vapor pressure deficit (VPD), (**c**) solar radiation (Rg), and (**d**) soil water content (SWC) (grey bars indicate the wet season).

Figure 5 shows the multiyear average values of key climatic variables. All the climatic variables exhibited strong seasonal trends. Air temperature (Ta) was highest in the wet season (Fig. 5a). VPD and solar radiation (Rg) showed similar seasonal patterns (Fig. 5b,c). Soil water content at 5 cm (SWC_5) exhibited strong seasonal trends with the highest values occurring during the wet season and lowest values occurring during the dry season (Fig. 5d). However, soil water content at 100 cm (SWC_100) showed weak seasonal patterns (Fig. 5d).

**Figure 6:**
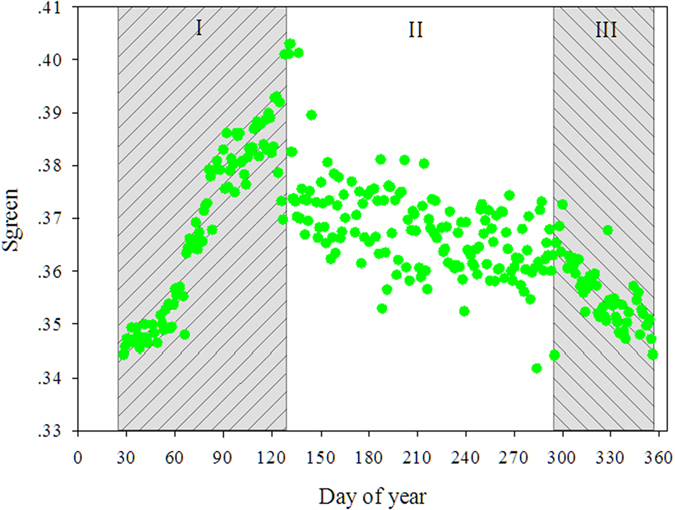
Green index values for the forest canopy. (I) leaf emergence stage, (II) leaf development stage, and (III) leaf senescence stage.

Figure 6 shows the seasonal variations in the camera-based green index of the canopy dominant tree species. The green index curve followed the seasonal course with an increase during the later dry season (DOY 30 to 130), a peak value in the early wet season with a subsequent slight decline, followed by a strong decline in the early dry season (DOY 295 to 355). To identify the WUE response to environmental controls in different phenophases, we selected three stages of canopy phenology: (I) leaf emergence stage, (II) leaf development stage, and (III) leaf senescence stage (Fig. 6 and Figure S2).

The correlation matrix and significance test between WUE and environmental variables at the three stages of canopy phenology are shown in Table 2. During the leaf emergence stage, the variation of WUE could be suitably explained by water-related variables (RH, SWC_100) and Sgreen. During the leaf development stage, the only significant correlation was found between WUE and two climatic variables (Ta and RH). During the leaf senescence stage, Rg exerted strong influence over WUE. At the annual scale, the variation of WUE could be mostly explained by the climatic variables and canopy phenology.

### Effect of drought stress on WUE

Rainfall decreased from September 2009 through the end of the dry season in 2010.

**Figure 7:**
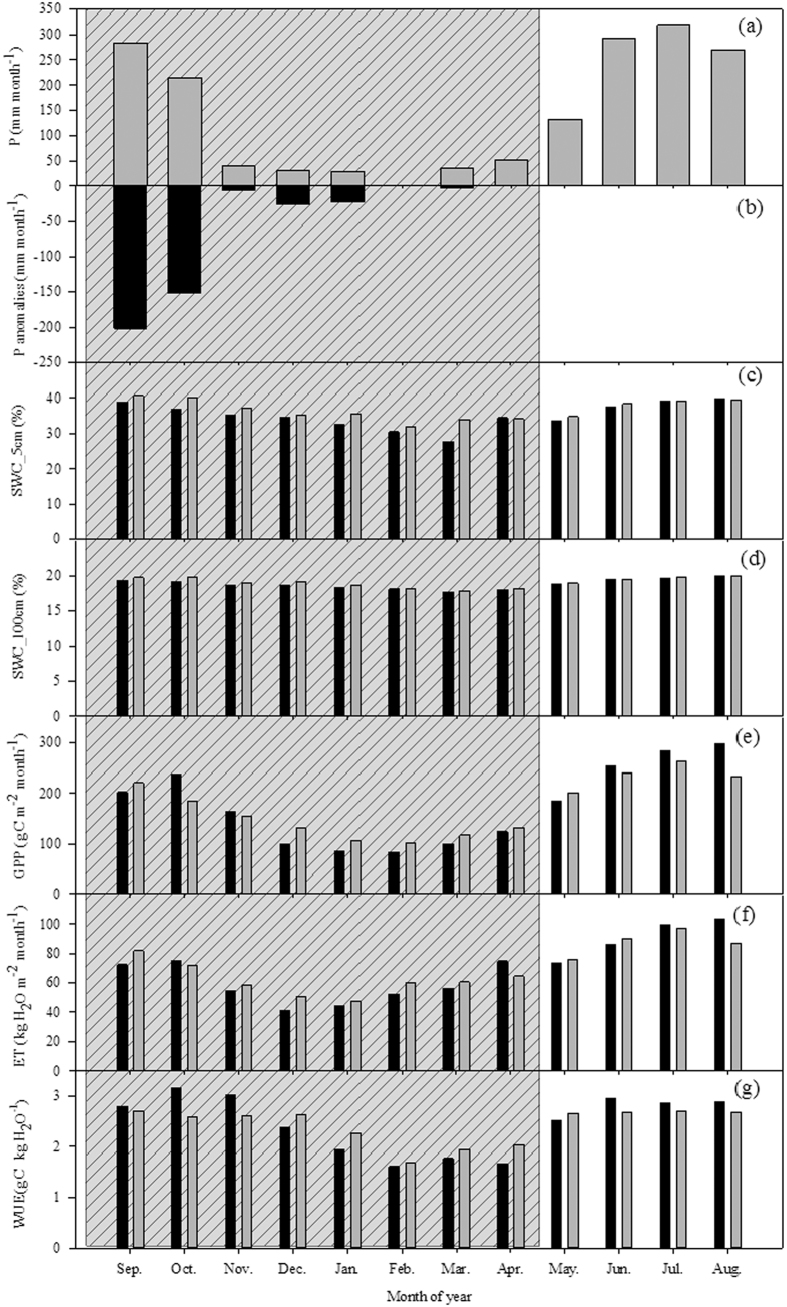
The impact of the 2009–2010 drought on ecosystem water use efficiency: (**a**) mean precipitation in normal years (2011–2013); (**b**) rainfall anomalies: precipitation in 2009–2010 minus mean precipitation; (**c**) soil moisture in 5 cm depth; (**d**) soil moisture in 100 cm depth; (**e**) gross primary production; (**f**) evapotranspiration; and (**g**) water use efficiency. Black bars represent values during September 2009 to April 2010, and grey bars indicate other normal year mean values.

The precipitation anomaly during 2009 was also reflected in the temporal patterns of soil moisture (Fig. 7), with a substantial decrease.

In general, WUE increased during the later wet season (September–November). From December to April, WUE was decreased during the drought year.

By examining the GPP and ET of each month (Fig. 7), it can be seen that increased WUE in September was caused by more decreased ET, and increased WUE in October was caused by more increased GPP. From December to March, GPP and ET were decreased, while reduced GPP was higher than ET, resulting in decreased WUE in this period. The WUE monthly pattern was determined by the shift of GPP and ET.

## Discussion

Subtropical evergreen forests represent the transition between temperate and tropical forests and are among the most important biomes on Earth because of their large primary productivity^28^ and their roles as hot spots of biodiversity^29^. They are widely spread and well protected in Yunnan province, southwestern China. The Ailaoshan Nature Reserve hosts about 5000 ha of primary subtropical evergreen forest. However, these forests are facing strong pressure related to human disturbance and global climate change; unfortunately, little is known about the dynamics of these processes.

The WUE of this subtropical evergreen forest was close to that of other subtropical forests, such as the Qianyanzhou evergreen forest (2.52 g C kg H_2_O^−1^) in China^6^ and a Florida evergreen forest (2.35 g C kg H_2_O^−1^) in the US^30^, while slightly higher than that in the Dinghushan evergreen broadleaved forest (1.88 g C kg H_2_O^−1^) in China^6^. In contrast, the WUE of this evergreen broadleaved forest was lower than that of temperate forests. These differences could partly be explained by the fact that subtropical forests with abundant water do not need to maintain metabolic functionality to prevent water loss.

From this study, we can see that WUE in this subtropical forest were highly variable at different time scales. Here we tried to explain the possible reasons for the patterns at different time scales (diurnal, daily, seasonal and annual scales).

At the diurnal scale, there was large variation in WUE during the wet and dry season, showing two peaks in the morning and afternoon, respectively. The evidence of these diurnal time changes might obey stomatal optimization principle^31^,^32^, which assumes that the regulatory role of stomata is to simultaneously maximize the carbon gain rate while minimizing the rate of water losses. In the early morning (08:00–08:30), VPD was small and solar radiation increased sharply (from 0 to 160 W m^−2^) (Figure S5). Therefore, the stomatal conductance of the whole canopy trees might also open during the short period, resulting in the canopy photosynthesis or carbon gain increased (Figure S6a). However, ET was still very low at the same time (Figure S6b). Therefore, WUE was high at the early morning. With the increasing of VPD and solar radiation (Figure S5), the stomatal conductance decreased gradually and ET increased. Especially, GPP declined at noon (12:00) (Figure S6a), due to the stomatal closure of the canopy trees probably. Previous study in this forest also showed that the fourteen broadleaf tree species significantly down-regulated leaf stomatal conductance at midday^33^. Meanwhile, ET kept high values in the afternoon (12:30–15:30) (Figure S6b). Therefore, WUE was low steady-state during this period. In the evening (18:00–18:30), canopy photosynthesis kept high level (9–11 μmol m^−2^ s^−1^) (Figure S6a). In contrast, ET decreased sharply at the same time (Figure S6b), resulting in the second peak of WUE.

VPD in the wet season was lower than that in dry season (Figure S5a). Consequently, the canopy stomatal conductance would be expected to be high and the tree photosynthesis was higher in the wet season. On the other hand, the decrease amplitude of ET (around 25%) in the dry season was less than that of GPP (around 80%). Overall, WUE in the wet season was always higher than that in the dry season, due to the more increased GPP in the wet season.

As demonstrated above, WUE was strongly depended on VPD at smaller time scales. Therefore, underling water use efficiency was proposed to incorporate the effects of VPD with reference to GPP-ET relationship via stomatal conductance. UWUE only reached a peak in the evening, in consistent with the pattern of VPD (Figure S5a).

At the daily time scale, GPP and VPD^0.5^ combination would lead to a nearly optimal linear relationship with ET (Figure S7), suggesting UWUE could be a suitable and superior formulation when applied to the daily time scale. This is consistent with the results from 42 AmeriFlux sites^34^. Conversely, a constant UWUE at daily time scale might be used to predict daily GPP using mean daily VPD and ET^34^.

At the seasonal scale, a sudden decrease of WUE was found during the late dry season (Fig. 3a). This may be explained by the lower GPP during this period (Fig. 7e). Some of the canopy trees shed their leaves in the late dry season to cope with the soil water deficit (Fig. 7c,d) and high atmospheric water demand (Fig. 5b).

Most previous studies have only focused on climatic drivers of WUE in different seasons^14^,^35^. Information about canopy phenology development is lacking. Like temperate and boreal forests, the impact of canopy phenology modification (leaf emergence, development, and senescence of some deciduous tree species) on carbon gain and water loss in subtropical forests is likely to also be important. In order to better understand the effect of phenology, canopy development needs be quantified.

Phenological information was derived from tower-based digital imagery from a standard RGB camera in the study forest (Figure S2). During the leaf emergence stage, the canopy green index had a strong positive impact on WUE (Table 2). In addition, Ta, Rg, and soil moisture in 5 cm were also the dominant driving forces of WUE, indicated by the negative correlation between the drivers and WUE. The environmental and biological factors regulated the WUE variation in this subtropical forest during the leaf emergence stage together. However, the correlation of WUE to canopy green index was weaker than to climatic variations during the leaf development and senescence stages. This might be explained by fact that the variations of vegetation phenology were relatively stable during the study period in this evergreen forest.

At an annual scale, WUE increased in the dry year (2009). This result was consistent with results in a mature boreal aspen stand^36^. Relative magnitudes of GPP and ET represented the sensitivity of different physical and biological processes to drought in different seasons and year^11^. Although ET decreased simultaneously during drought periods, its response was larger than the GPP response, leading to a higher WUE in the dry year. A striking 10% increase in WUE (Fig. 4) in 2009 was associated with a 12% decrease observed in annual ET during the drought period. More cloudy days in 2009 were observed. Possible causes for this pattern are: (1) Clouds and fog may reduce the incoming solar radiation but increase the relative proportion of diffuse radiation in the forest (Figure S8). In addition, increases in diffuse PAR fraction, which is produced when clouds interact with and scatter incoming solar radiation, may be even more beneficial than equal increases in direct light^37^. Diffuse light can penetrate deeper into the subtropical evergreen broadleaved forest canopy and reach lower canopy leaves that would normally be light-limited on clear days^38^. (2) On the other hand, this primary subtropical broad-leaved forest in our study had a stand age >300 years and was free of management^28^. Most dominant tree species in the evergreen forest might have a more conservative water and deeper root systems. The impact of water deficit on growth may much less in the mesic site and have a high growth plasticity in response to short drought stress. (3) However, ET is an integrated product of soil–plant–atmosphere interactions and involves direct evaporation from the soil surface (Es), canopy evaporation of intercepted cloud or precipitated water (Ei), and plant transpiration (Et)^39^. In this site, Es decreased (29%) more significantly than Et (12%) during the beginning period of drought (unpublished data), resulting in the total ET decreased in 2009 significantly. Overall, annual GPP responded less to drought stress than ET in 2009.

The response of WUE to drought in different ecosystems might be of a different magnitude or even of completely different directions. For example, although both occur in Yunnan province, Southwest China, our study site in the subtropical evergreen forest is different from a tropical rainforest, which was no significant difference between WUE during the drought and the 9-year mean values^14^. WUE of most European CARBONEUROPE/FLUXNET monitoring sites decreased during the year of the heatwave, due to a larger decline in GPP than in ET^40^. These results revealed large variation in WUE in the different forests, highlighting the importance of individual site characteristics in determining the extent to which WUE may be more controlled by GPP or ET dynamics during the drought period.

Our findings highlight the complexities involved in climatic and biotic factors in the different time scales. Future climate warming is likely to affect the CO_2_/H_2_O exchange and the phenology. Furthermore, a warmer and drier regional climate in montane cloud belts is likely to cause an increase in the lifting condensation level^41^, which potentially leads to a reduction of fog density or fog frequency^42^. This would exhibit a strong influence on the eco-physiological factors and processes in the cloud forests^43^. Ongoing research at the study site should look closely at different conditions (tree species, ecophysiology, fog, *et al*.) in order to better study the forest responses induced by climate change at different scales.

## Methods

### Ecosystem water use efficiency (WUE)

WUE is defined as WUE = GPP/ET.

Where, GPP is gross ecosystem primary productivity (g C m^−2^ time^−1^), and ET is evapotranspiration (kg H_2_O m^−2^ time^−1^).

GPP can be expressed as


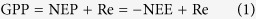


Where, NEE is the net ecosystem CO_2_ exchange (g C m^−2^ time^−1^), the balance between photosynthetic C assimilation and C-releasing respiration, Re is total ecosystem respiration (g C m^−2^ time^−1^), and NEP is net ecosystem productivity (g C m^−2^ time^−1^), which is equal to negative NEE. NEE or NEP can be directly measured by EC techniques.

### The underlying water use efficiency (UWUE)

The underlying water use efficiency (g C hPa kg H_2_O^−1^) is suitable for estimating ecosystem GPP and ET and identifying the photosynthesis/transpiration balance and water use efficiency variations among different vegetation types over the long term^44^.


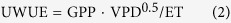


Where, VPD is the water vapor pressure deficit (hPa).

The dependence of UWUE on environmental conditions indicates possible adaptive adjustment of ecosystem physiology in response to a changing environment. The UWUE is more appropriate than WUE for describing the biochemical functions of plants^34^,^44^.

UWUE will represent different temporal resolutions in the following sections depending on the underlying timescale of interest, either daily sums of GPP and ET, and mean daylight VPD on a daily scale, or integrated GPP and ET on an annual scale.

### Microclimatic Observations

An eddy covariance system was installed on a scaffolding tower at a height of 34 m above ground level, which is 14 m above the mean canopy height. The flux system consisted of a 3-D sonic anemometer (CSAT3, Campbell Scientific Inc., Logan, UT, USA) and an open-path infrared gas analyzer (Li-7500, Li-Cor Inc., Lincoln, NE, USA). The operation of the two instruments and the recording of raw data at 10 Hz were controlled by a datalogger (CR3000, Li-Cor Inc.). Instruments for measuring air temperature and air humidity (HMP45C, Vaisala, Helsinki, Finland) as well as wind speed (A100R, Vector Instruments, Denhighshire, UK) were installed at seven heights and were recorded at 30-min intervals. Instruments for measuring wind direction (W200P, Vector Instruments) were installed at the top of the tower (34 m). Radiation sensors for downward and upward, short and long-wave radiation (CNR-1/CM11, Kipp & Zonen, Delft, the Netherlands) were installed at 26 m height on a horizontal pole 3 m away from the tower. Further, photosynthetically active radiation (PAR) was measured using linear sensors (LQS70-10, Apogee, USA). Profiles of soil temperature and soil moisture were measured at 0, 5, 10, 15, 20, 40, 60, and 100 cm depths (CS616_L, Campbell, USA; and 105T/107L, Campbell, USA, respectively). Soil heat flux was measured by two heat flux plates (HFP01, HukseFlux, Netherlands).

The micrometeorological measurements were started in August 2008 and have been maintained continuously since then.

### Data processing

Carbon flux, latent heat flux (LE) and sensible heat flux (H) were computed using an eddy flux data-processing program from 30-min time periods^45^. To obtain daily, monthly, and annual sums, it was necessary to perform a gap-filling routine. Generally, data gaps occurred at night when precipitation obscured the sensors, or due to shortages in the power supply. Three-dimensional rotation of the coordinates was applied to all half-hour wind components to remove the effect of instrument tilt and irregularity of airflow. The flux data were corrected for the variation of air density caused by density fluctuations due to heat and water vapor fluxes^46^.

The energy balance closure quantifies the ratio to which the sum of the fluxes of sensible heat and latent heat use up the energy that is provided by the radiation balance (Rn) minus the soil heat flux (G). Overall, the energy closure ratio was 0.70 using 30-min data (Figure S3). This is good within the range of values found at other FLUXnet sites^47^. For the purpose of further data analysis of ET, we accounted for the non-closure of the energy balance by applying corrections with the buoyancy flux ratio^48^. The eddy-covariance-based flux footprint mostly covered the subtropical forest canopy (Figure S4).

The missing data in flux and micrometeorological measurements were filled using an online program based on standardized methods after Reichstein *et al*.^45^ maintained by the Max Planck Institute (Germany) (www.bgc-jena.mpg.de/~MDIwork/eddyproc/index.php). Gaps in the precipitation data subset were filled using data from a nearby weather station.

## Additional Information

**How to cite this article:** Song, Q.-H. *et al*. Water use efficiency in a primary subtropical evergreen forest in Southwest China. *Sci. Rep.* **7**, 43031; doi: 10.1038/srep43031 (2017).

**Publisher's note:** Springer Nature remains neutral with regard to jurisdictional claims in published maps and institutional affiliations.

## Supplementary Material

Supplementary Material

## Figures and Tables

**Table 1:** Annual mean GPP, ET, Water vapor pressure deficit (VPD), WUE and UWUE in the five years.

Year	GPP g C m^−2^ year^−1^	ET kg H_2_O m^−2^ year^−1^	VPD hPa	WUE g C kg H_2_O^−1^	UWUEg C hPa^0.5^ kg H_2_O^−1^
2009	2106	785	3.14	2.68	4.76
2010	2315	905	3.15	2.56	4.54
2011	2214	864	3.17	2.56	4.56
2012	2092	901	3.36	2.32	4.26
2013	1966	862	3.68	2.28	4.38

**Table 2:** Pearson’s correlation between variables and water use efficiency in the different stages.

	Leaf emergence	Leaf development	Leaf senescence	Whole year
T_a_	−0.019	0.421^**^	−0.225	0.140^**^
RH	0.675^**^	−0.336^**^	0.483^**^	0.645^**^
Rg	−0.585^**^	0.226	−0.484^**^	−0.523^**^
SWC_5	−0.204	−0.082	−0.237	0.206^**^
SWC_100	0.414^**^	−0.184	−0.052	0.485^**^
P	0.223	−0.168	−0.191	0.025
Sgreen	0.385^**^	0.078	0.102	−0.019^*^

Single and double asterisks indicate statistical significance at the 0.05 and 0.01 levels, respectively.
