# Supplementary material for: Water use efficiency in a primary subtropical evergreen forest in Southwest China
Source: Sci Rep. 2017 Feb 20;7:43031. doi: 10.1038/srep43031 (PMC5316949; doi:10.1038/srep43031)
Supplement: Supplementary Material [file srep43031-s1.pdf]

Supplementary information for:

Water use efficiency in a primary subtropical evergreen forest in Southwest China

Qing-Hai Song[1,2], Xue-Hai Fei[1,2,4], Yi-Ping Zhang[1,2,3*], Li-Qing Sha[1,2], Yun-Tong Liu[1,2], Wen-Jun Zhou[1,2], Chuan-Sheng Wu[1,2,4], Zhi-Yun Lu[1,3], Kang Luo[1,3,4], Jin-Bo Gao[1,2,4], Yu-Hong Liu[1,3]

[1] Key Laboratory of Tropical Forest Ecology, Xishuangbanna Tropical Botanical Garden, Chinese Academy of Sciences, Menglun 666303, China

[2] Global Change Ecology Group, Xishuangbanna Tropical Botanical Garden, Chinese Academy of Sciences, Menglun 666303,China

[3] Ailaoshan Station for Subtropical Forest Ecosystem Studies, Jingdong 676200, Yunnan, China

[4] University of Chinese Academy of Sciences, Beijing 100049, China

*Correspondence and requests for materials should be addressed to Y.P.Z.(email: yipingzh@xtbg.ac.cn)

The flux tower

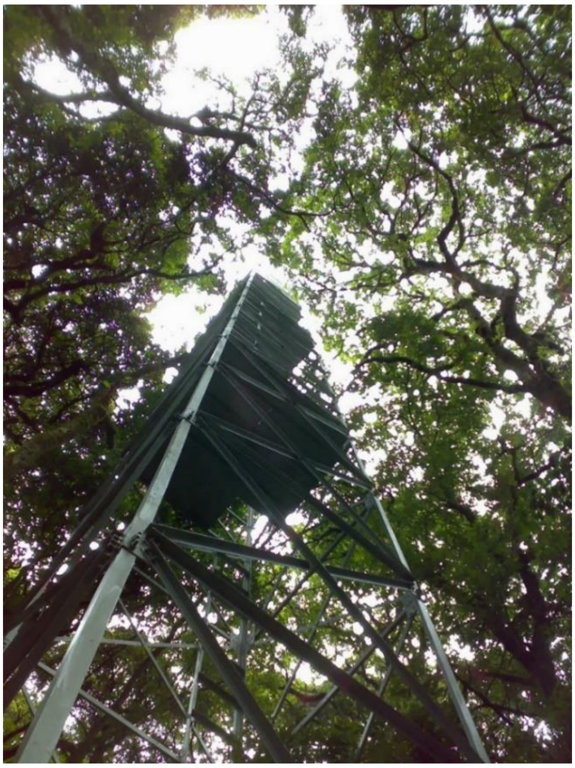

Figure S1. The flux tower in the subtropical evergreen forest (This picture was taken by Qing-Hai Song)

The canopy pictures in the different stages

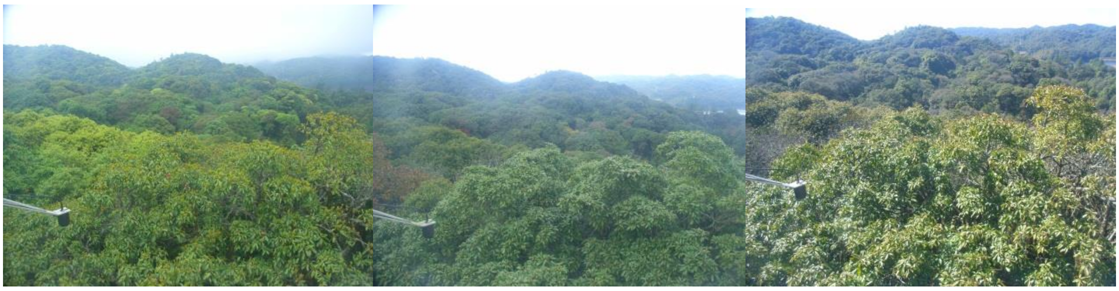

Figure S2. Sample images (record on 21 Feb., 6 May. and 4 Dec. 2011) from the camera on the forest canopy. These pictures represented three stages of canopy phenology: (I) leaf emergence stage, (II) leaf development stage, and (III) leaf senescence stage.

Energy balance closure test

The energy balance closure quantifies the ratio to which the sum of the fluxes of sensible heat and latent heat use up the energy that is provided by the radiation balance (Rn) minus the soil heat flux (G). Overall, the energy closure ratio was 0.70 using 30-min data (Figure S3). This is good within the range of values found at other FLUXnet sites[1]. For the purpose of further data analysis of ET, we accounted for the non-closure of the energy balance by applying corrections with the buoyancy flux ratio [2].

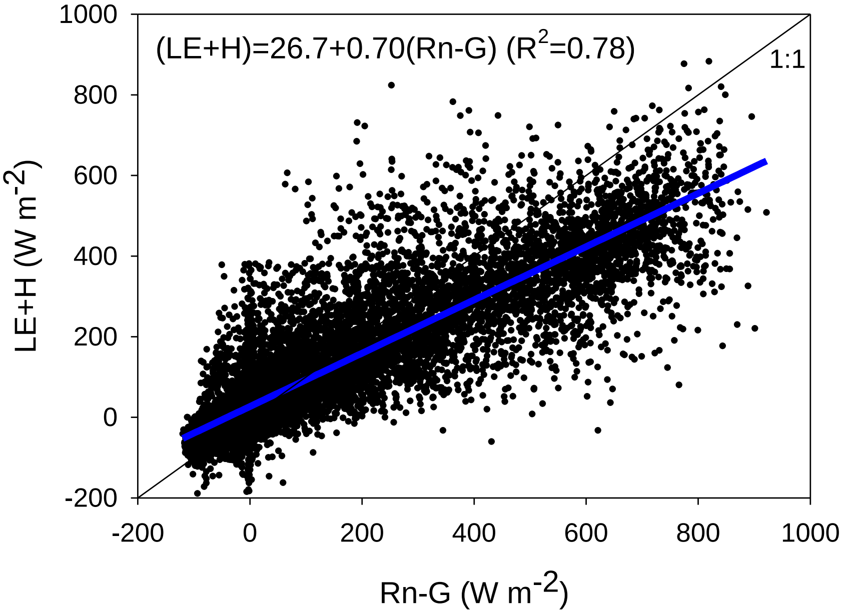

Figure S3. Energy balance scatter plot showing degree of energy balance closure based on 30-min raw data.

Footprint

The micrometeorological measurements were started in August 2008 and have been maintained continuously since then. Sensor calibrations of the LI-7500 (Li-Cor Inc., Lincoln, NE, USA) have been performed every two years to ensure accurate concentration measurements. For the calculation of the flux source area, the Lagrangian backward footprint model according to Kljun *et al.*, (2004)[3] was used, which requires the input variables z_m/z_0, z_m/L, and Sv/u^*. Measurement height z_m was calculated from roughness length (z_0) and zero-plane displacement, L is the Monine Obukhov length, Sv is the variance of horizontal wind direction. The eddy-covariance-based flux footprint mostly covered the subtropical forest canopy (Figure S4).

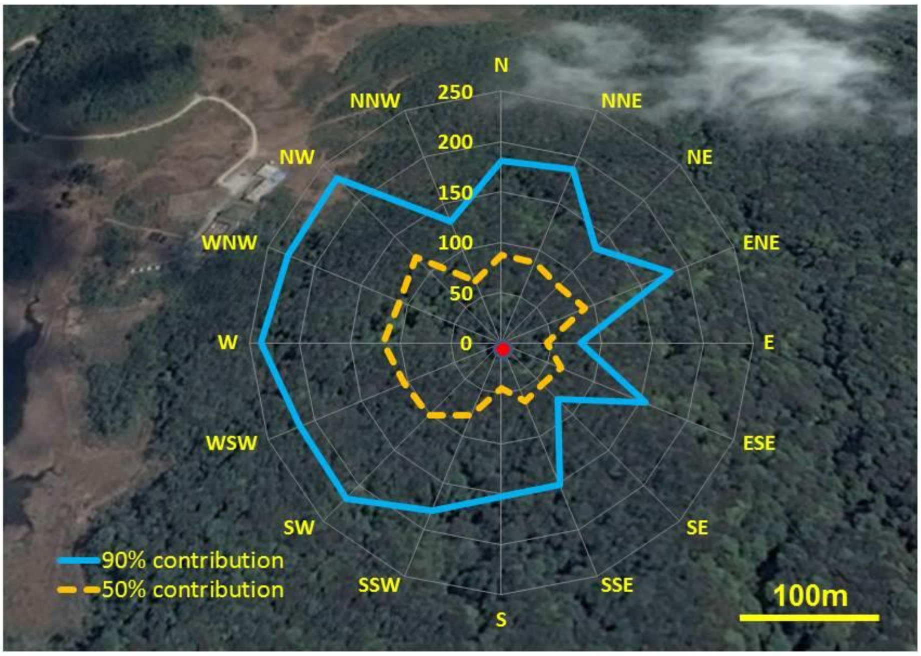

Figure S4. Satellite picture of the study site (April 10, 2014, from Google Earth). The blue and yellow lines represent the maximum extent of the 90 % and 50 % footprint distances under stable condition, respectively. Red point is the flux tower. Footprint parameterization from Kljun *et al.*, (2004).

The diurnal patterns of GPP, ET and VPD

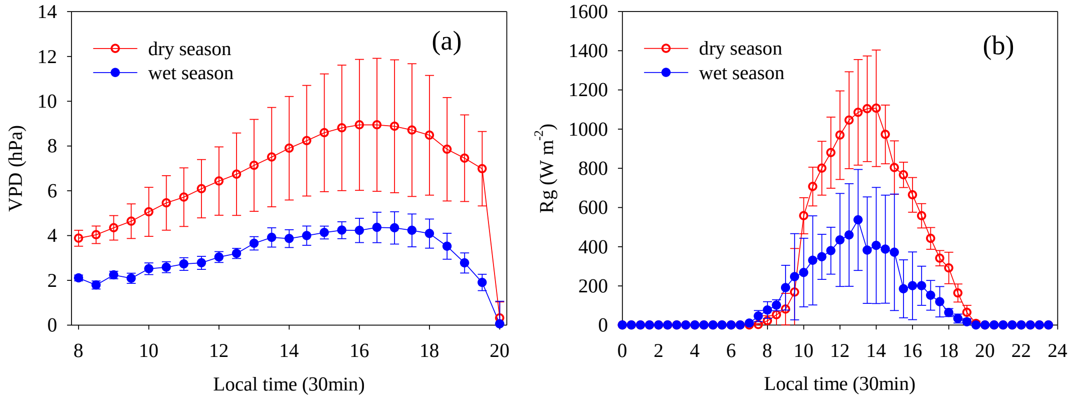

Figure S5. The diurnal dynamics of VPD (a) and Rg (b) during the wet and dry season.

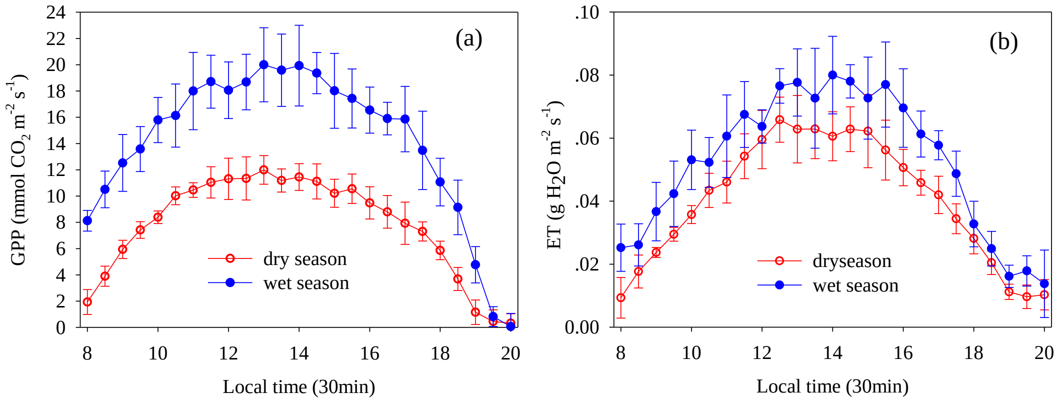

Figure S6. The diurnal dynamics of GPP and ET during the wet and dry season.

Daily underling water use efficiency (UWUE)

We selected two days in dry season (Apr. 15, 2009) and wet season (Aug. 10, 2009), respectively, to analysis the relationship between ET and GPP*VPD$^{0.5}$.

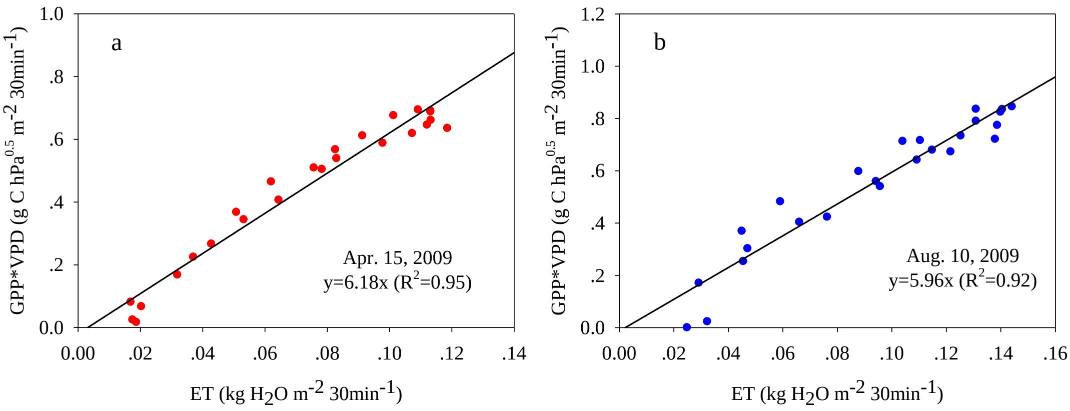

Figure S7. Diurnal relationships between ET and GPP*VPD$^{0.5}$.

Fog frequency in this forest

Visibility was measured by PWS 100 (Campbell Scientific Inc., Logan, UT, USA) in this forest. Conditions with visibility <1000 m were considered to be fog according to the World Meteorological Organization (WMO) definition. The fog deposition occurs frequently in the whole year in this forest (Figure S8).

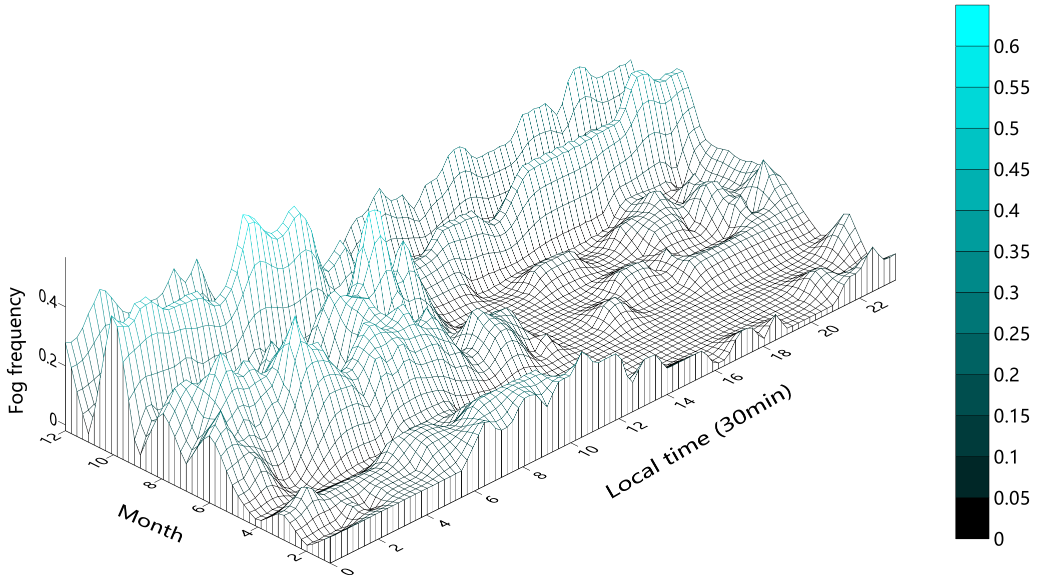

Figure S8. Fog frequency in this subtropical forest.

References

1. Wilson, K. *et al*. Energy balance closure at FLUXNET Sites. *Agr. Forest Meteorol*. **113(1–4)**, 223–243 (2002).
2. Charuchittipan, D., Babel, W., Mauder, M., Leps, J.P. & Foken, T. Extension of the averaging time in eddy-covariance measurements and its effect on the energy balance closure. *Boundary Lay. Meteorol*. 152 (3), 303-327 (2014).
3. Kljun, N., Calanca, P., Rotach, M.W. & Schmid, H.P. A simple parameterization for flux footprint predictions. *Boundary Lay. Meteorol*. **112** (3), 503–523 (2004).
